# A Reversibly Responsive Fluorochromic Hydrogel Based on Lanthanide–Mannose Complex

**DOI:** 10.1002/advs.201802112

**Published:** 2019-03-13

**Authors:** Ke Meng, Chi Yao, Qianmin Ma, Zhaohui Xue, Yaping Du, Wenguang Liu, Dayong Yang

**Affiliations:** ^1^ Frontier Science Center for Synthetic Biology and Key Laboratory of Systems Bioengineering (MOE) School of Chemical Engineering and Technology Tianjin University Tianjin 300350 P. R. China; ^2^ School of Materials Science and Engineering & National Institute for Advanced Materials Center for Rare Earth and Inorganic Functional Materials Nankai University Tianjin 300350 P. R. China; ^3^ School of Materials Science and Engineering Tianjin Key Laboratory of Composite and Functional Materials Tianjin University Tianjin 300350 P. R. China

**Keywords:** 3D cell culture, fluorochromic materials, hydrogels, lanthanide–mannose complex, responsive materials

## Abstract

Fluorochromic materials that are dynamic in response to external stimuli are of great interest for the development of advanced sensors and luminescent materials. Herein, a design based on a lanthanide‐containing polymeric hydrogel possessing characteristic emission of lanthanides (Eu and Tb) and showing response to stimuli of metal ions is reported. The fluorochromic hydrogel is prepared using a lanthanide–mannose complex in gelation matrix. The lanthanide–mannose complex shows tunable fluorescent emission in response to Fe^2+^, due to the inhibition of the “antenna effect” between metal ions and ligands upon stimulation. The fluorescent hydrogel shows reversible “On/Off” fluorochromic response to Fe^2+^/ethylenediaminetetraacetic acid (EDTA). Remarkably, the fluorescent hydrogel is proven nontoxic and biocompatible; and a proof‐of‐application as in situ 3D cell culture extracellular matrix with reversible fluorochromic “On/Off” switch upon Fe^2+^/EDTA is demonstrated. This reversibly responsive fluorochromic hydrogel demonstrates a way to fabricate smart optical materials, particularly for biological‐related applications where reversible response is required.

## 1 Introduction

Adaptive optical materials that can adapt or actuate in response to external stimuli such as light, temperature, pH, metal ions, mechanical stress, and chemical stress are critical to the development of smart material systems.^1^ In recent years, there are profound interests to develop new fluorochromic materials (light emission) for a broad range of applications in probes, sensors, indicators and display devices.^2^ One typical fluorochromic materials are organic chromophores such as spirothiopyran and diethyl‐2,5‐bis(4‐(trifluoromethyl)phenylamino)terephthalate, which showed isomerization and changeable conformations upon external stimuli such as mechanical force and temperature, resulting in fluorescence change.^3^ Another important and promising fluorochromic materials are lanthanide (e.g., Eu and Tb) based materials by virtue of the advantageous performance such as long luminescence lifetime, and sharp emission bands.^4^ As lanthanide ions coordinate with ligands, ligands act as an “antenna” to absorb energy and then transfer the energy to lanthanide ions, which result in characteristic luminescence of lanthanide.^5^ The fluorescence change of organic chromophores upon stimuli relies on the breakage of covalent bonds; in contrast, the fluorescence of lanthanide/ligand complexes is more sensitive to stimuli due to the dynamic noncovalent coordination between lanthanide and ligands.[[qv: 5c,6]]

Most lanthanide‐based materials were synthesized in organic solvents, which restricted the applications, in particular in biomedical engineering. Instead, water is considered as the most environmentally friendly solvent, which has been extensively used as media to synthesize biocompatible materials.^7^ Moreover, water associating with molecular network generates three‐dimensional networked materials, known as hydrogels, which are increasingly applied in a variety of important applications such as tissue engineering due to their high water content, biocompatibility, and the gel‐like character.^8^ Recently, the preparation of lanthanide‐based hydrogels has been successfully demonstrated.[[qv: 1g,9]] For example, Gunnlaugsson and co‐workers prepared lanthanide–cyclen hydrogels and demonstrated application in diagnosis of urinary tract infection.[[qv: 9b]] He and co‐workers synthesized lanthanide–iminodicetate hydrogels which showed fluorochromic response to multistimuli.[[qv: 1g]] Li and co‐workers reported lanthanide luminescent hybrid hydrogels which possessed reversible phase transition upon photoirradiation.^10^

Herein we create new lanthanide‐containing polymeric hydrogels possessing characteristic luminescence of lanthanides (Eu and Tb) and showing response to stimuli of metal ions. The fluorochromic hydrogel is prepared by using lanthanide–mannose complex in gelation matrix. It is noteworthy that both mannose and gelatin are biomolecules from life systems which ensures the nontoxicity and biocompatibility. Lanthanide–mannose complex shows tunable fluorescent emission upon Fe^2+^, due to the inhibition of the “antenna effect” between metal ions and ligands upon stimuli. The luminescent hydrogel shows reversible “On/Off” fluorochromic response to Fe^2+^/EDTA. Remarkably, the luminescent hydrogel is proven nontoxic and biocompatible; and a proof‐of‐application as in situ 3D cell culture extracellular matrix with reversible fluorochromic “On/Off” switch upon Fe^2+^/EDTA for high contrast observation is demonstrated.

## 2 Results and Discussion

**Figure 1:**
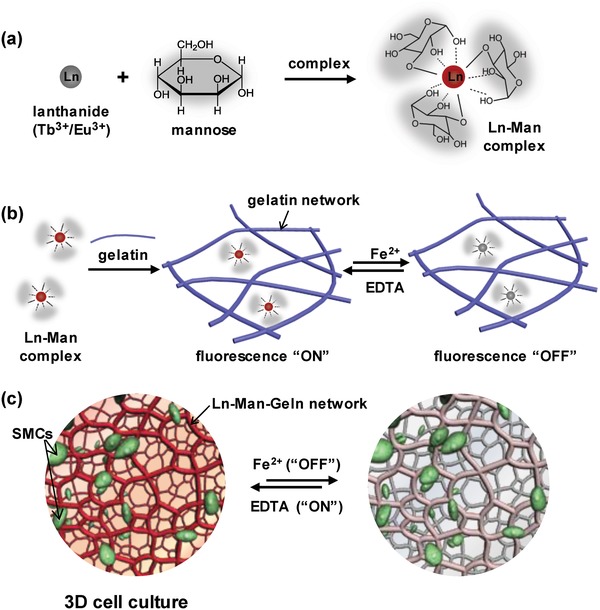
a) Scheme of the formation of lanthanide–mannose (Ln–Man) complex through the coordination of mannose with lanthanide ions (Tb^3+^/Eu^3+^). b) Scheme of the formation of lanthanide–mannose–gelatin (Ln–Man–Geln) hydrogel by introducing Ln–Man into gelatin network, and the property of fluorescence “On/Off” upon Fe^2+^/EDTA. c) Illustration of the Ln–Man–Geln hydrogel as a 3D cell culture matrix for reversible fluorochromic “On/Off” switch upon Fe^2+^/EDTA.

Some early works have demonstrated that saccharide could form complexes with lanthanide mostly in organic solvents.^11^ In our design strategy, we utilized saccharides as a category of ligands to coordinate with lanthanide ions (Eu^3+^ and Tb^3+^) and formed fluorescent Ln–Sac complexes in water (**Figure** **1**a). Then, we incorporated Ln–Man complex into gelatin molecular network, realizing the formation of lanthanide–mannose–gelatin (Ln–Man–Geln) hydrogels for the reversible fluorescence “On/Off” switch response to stimuli (Figure 1b). And the Ln–Man–Geln hydrogels can as a 3D cell culture matrix to realize reversible “On/Off” fluorescence of matrix during the observation of cell culture system (Figure 1c).

**Figure 2:**
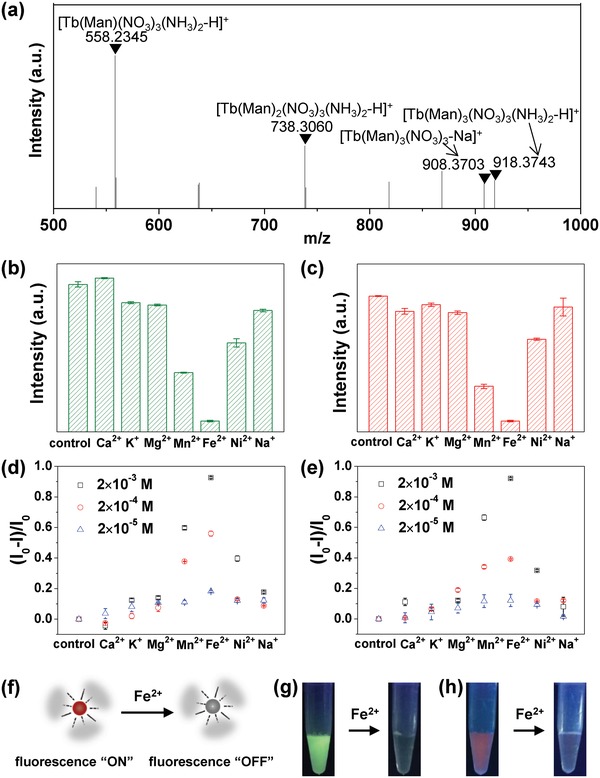
Ln–Man complex. a) MALDI‐MS spectrum of Tb–Man complex. b,c) Fluorochromic response of Tb–Man complex b) and Eu–Man complex c) upon different metal ions. The concentration of metal ions was 2 × 10^−3^ m. The quenching efficiency of d) Tb–Man complex and e) Eu–Man complex upon different metal ions with varied concentrations. f) The illustration of the fluorescence “On/Off” response of Ln–Man upon Fe^2+^. g,h) Fluorescence images of g) Tb–Man complex and h) Eu–Man complex upon Fe^2+^ under 312 nm UV light. The concentration of Fe^2+^ was 2 × 10^−3^ m.

Herein we explored five typical monosaccharides including glucose, sorbose, galactose, fructose, and mannose for the formation of Ln–Sac complexes (Figure S1, Supporting Information), and tested their performance of fluorescence with varying the concentration of saccharides. All of them had a characteristic green emission of Tb^3+^ at λ = 545 nm (^5^D_4_ → ^7^F_5_) upon 312 nm UV light irradiation. Ln–Man complexes had relatively high fluorescence intensity and as the concentration of mannose increased, the fluorescence intensity of Ln–Man complexes increased first, and tended to be stable (Figure S2 and Figure S3, Supporting Information). We therefore chose mannose as the ligand to coordination with lanthanide ions, and the concentrations of mannose and lanthanide ions were optimized. Fourier transform infrared (FTIR) spectroscopy was performed to study the molecular interactions between mannose and lanthanide ions (Figure S4, Supporting Information). Upon the formation of Tb^3+^‐mannose (Tb–Man) complex, the peaks of mannose at 3433 (ν_‐OH_), 1417 (ν_‐CH_), and 1066 cm^−1^ (ν_‐CO_) shifted to 3417, 1384, and 1064 cm^−1^, and the peak of mannose at 2935 cm^−1^ (ν_‐OH_) produced two peaks at 2972 and 2975 cm^−1^; upon the formation of Eu^3+^‐mannose (Eu–Man) complex, the peaks of mannose at 3433 (ν_‐OH_), 1417 (ν_‐CH_), and 1066 cm^−1^ (ν_‐CO_) shifted to 3423, 1641, 1384, and 1068 cm^−1^, and the peak of mannose at 2935 cm^−1^ (ν_‐OH_) produced two peaks at 2976 and 2920 cm^−1^. And from the matrix assisted laser desorption/ionization mass spectrometry (MALDI‐MS) spectrum of Tb–Man complex and Eu–Man complex, the lanthanide ions are able to coordination with three mannose ligands simultaneously (**Figure** **2**a; Figure S5, Supporting Information). The Ln–Man complexes exhibited characteristic emission of Ln^3+^ upon the irradiation of 312 nm UV light: Tb–Man complex exhibited strong green emission at λ = 545 nm (^5^D_4_ → ^7^F_5_) (Figure S6a, Supporting Information), and Eu–Man complex exhibited strong red emission at λ = 615 nm (^5^D_0_ → ^7^F_2_) (Figure S6b, Supporting Information). The fluorescent excitation spectra of Tb–Man complex and Eu–Man complex showed a broad band in the range of 300–325 nm, which showed the occurrence of ligand to lanthanide ions energy transfer (Figure S6c,d, Supporting Information). The luminescent lifetime (τ) of Tb–Man complex and Eu–Man complex was 1.16 and 0.41 ms respectively (Figure S7, Supporting Information).

We next examined the fluorochromic response of Ln–Man complexes upon metal ions by considering the competitive coordination with mannose between lanthanide ions and other metal ions. We investigated seven typical metal ions including Ca^2+^, K^+^, Mg^2+^, Mn^2+^, Fe^2+^, Ni^2+^, and Na^+^, and varied the concentrations of metal ions (Figure 2b,c; Figure S8, Supporting Information). With the addition of other metal ions into Tb–Man or Eu–Man complex, the fluorescence of Ln–Man complexes was quenched in different degrees. Fe^2+^ showed the most remarkable quenching effect on the fluorescence of both Tb–Man and Eu–Man complexes (Figure 2b–e). During this process of fluorescence quenching, Fe^2+^ competed with Ln^3+^. Fe^2+^ as transition metal ion can provide more empty tracks to accept electronics of mannose; as a result, the “antenna effect” between mannose and lanthanide was weakened, and the emission intensity was strongly quenched.^12^ Metal ions (M*^n^* ^+^) combine with ligand (L) to form complex (ML*_n_*), and the equation is given as below

(1) $$M\;+\;nL\;\to \;{\mathrm{ML}}_{n}$$

The stability constant of complex is formulated as follows

(2) $$K\;=\;\frac{\left[{\mathrm{ML}}_{n}\right]}{\left[M\right]{\left[L\right]}^{\;n}}$$

$K_{{\mathrm{Fe}}^{2+}}$ is larger than the stability constant of other metal ions (Ca^2+^, K^+^, Mg^2+^, Mn^2+^, Ni^2+^, and Na^+^), and as a result Fe^2+^ showed the most quenching effect on the fluorescence of Ln–Man than other metal ions. Furthermore, with the increase of the concentration of Fe^2+^, the fluorescence intensity of Tb–Man and Eu–Man complexes gradually decreased (Figure S9, Supporting Information). With this mechanism, we presented the feature of fluorescence “On/Off” response upon Fe^2+^ and the illustration was shown in Figure 2f. Tb–Man and Eu–Man complexes that originally exhibited distinctive green and red fluorescence respectively, was quenched to colorless state when the Fe^2+^ ion in the final complexes was 2 × 10^−3^ m (Figure 2g,h).

**Figure 3:**
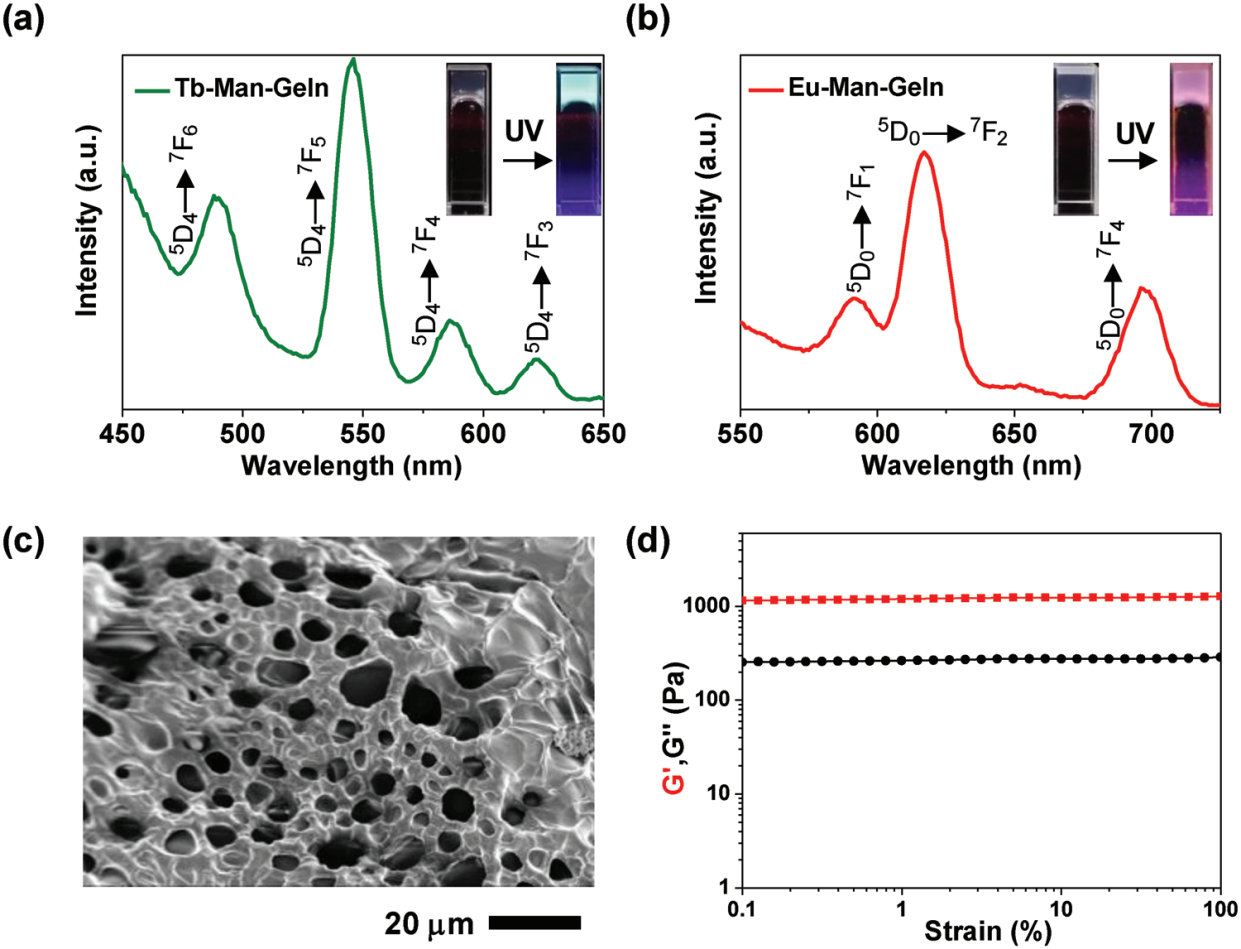
Ln–Man–Geln hydrogels. The fluorescent spectra and images of a) Tb–Man–Geln and b) Eu–Man–Geln hydrogels under daylight and UV light (312 nm). c) SEM image of Tb–Man‐Geln. d) The rheology properties of Tb–Man–Geln.

We further incorporated Ln–Man into gelatin molecular network, realizing the formation of Ln–Man–Geln hydrogels for the reversible fluorescence “On/Off” switch response to stimuli. To construct the effective fluorescence and response system of hydrogel, we optimized the content of gelatin to 18 wt% according to the fluorescence intensity (Figure S10, Supporting Information). Tb–Man–Geln hydrogel exhibited characteristic green emission at λ = 545 nm, and Eu–Man–Geln hydrogel exhibited characteristic red emission at λ = 615 nm upon 312 nm UV light excitation (**Figure** **3**a). The Tb–Man–Geln and Eu–Man–Geln hydrogels showed well‐distributed green and red fluorescence under fluorescence microscope, respectively (Figure S11, Supporting Information). The microstructures of Tb–Man–Geln and Eu–Man–Geln hydrogel were further investigated by scanning electronic microscopy (SEM), which exhibited a typical porous structure (Figure 3c). We then studied the mechanical properties of Tb–Man–Geln and Eu–Man–Geln hydrogels with a rotational rheometer. Rheology data confirmed the formation of hydrogel, as the shear‐storage modulus (*G*′) was constantly higher than the shear‐loss modulus (*G*″) (Figure 3d), showing similar mechanical strength to the hydrogel without complexes (pure gelatin, Figure S12a, Supporting Information). Gel stability of Tb–Man–Geln and Eu–Man–Geln was also confirmed via strain sweep mode. The *G*′ of Eu–Man–Geln was dominant over *G*″ throughout the strain range before 55.8%, indicating that the hydrogel was stable in this strain range (Figure S12b, Supporting Information). The luminescent lifetime (τ) of Tb–Man–Geln hydrogel and Eu–Man–Geln hydrogel was 1.18 and 0.42 ms respectively (Figure S13, Supporting Information). Compared with the luminescent lifetime (τ) of Tb–Man complex and Eu–Man complex, these results suggested that the luminescence can be well retained in gelation hydrogels.

**Figure 4:**
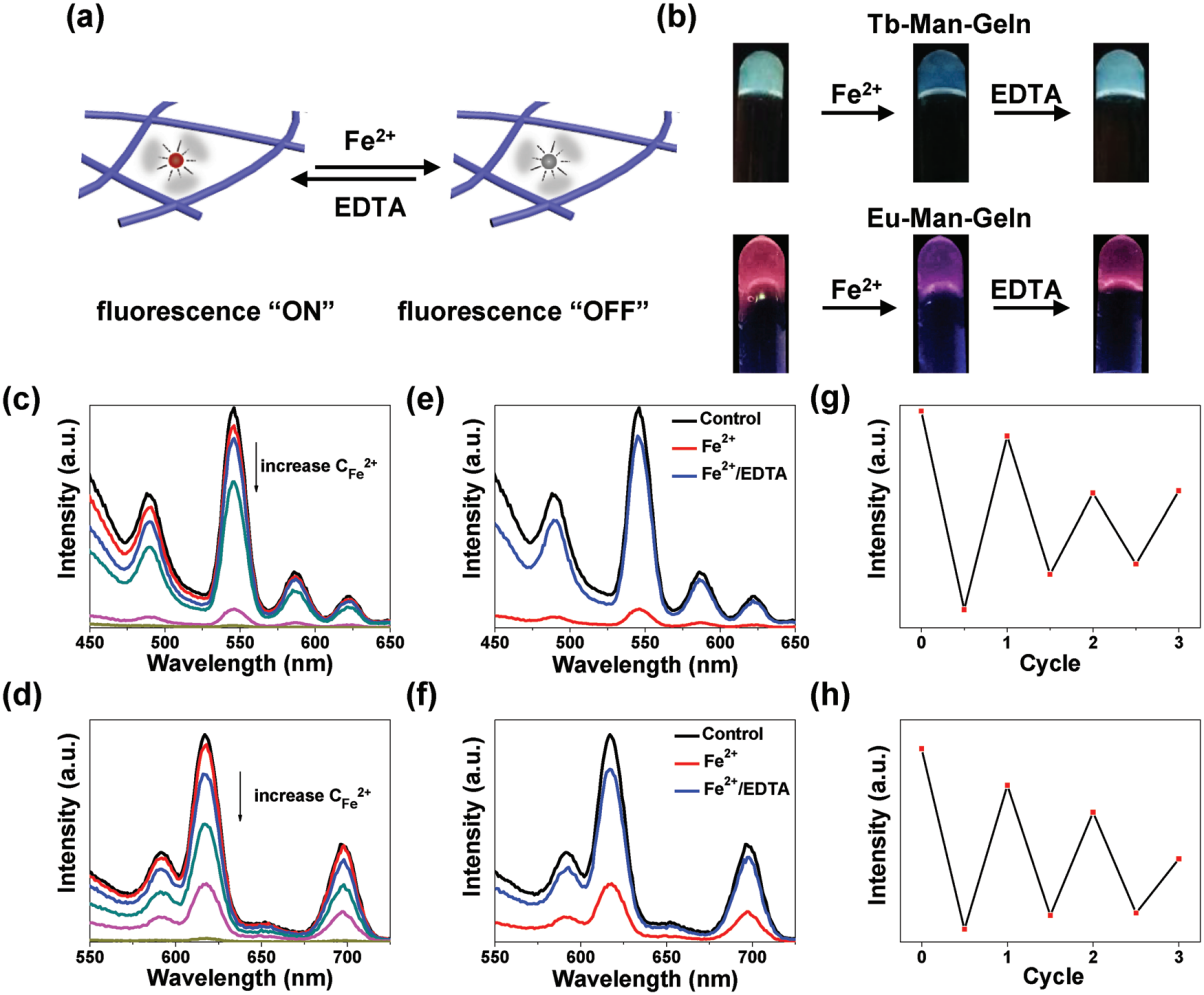
Reversible response of Ln–Man–Geln hydrogels upon Fe^2+^/EDTA. a) Illustration of the Ln–Man–Geln hydrogels as matrices for reversible fluorochromic “On/Off” switch upon Fe^2+^ ions. b) Photographs of the fluorescence “On/Off” of Tb–Man–Geln and Eu–Man–Geln upon UV light (312 nm) irradiation. Gradual decrease in the fluorescence intensity of c) Tb–Man–Geln and d) Eu–Man–Geln by increasing the concentration of Fe^2+^. Fluorescent spectra of the hydrogel, the hydrogel treated with Fe^2+^, and the recovered hydrogel treated with EDTA about e) Tb–Man–Geln and f) Eu–Man–Geln. Fluorescence quenching and recovery cycles of g) Tb–Man–Geln and h) Eu–Man–Geln.

After the successful construction of the Ln–Man–Geln hydrogel systems, we tested the fluorochromic response property of the hydrogels upon metal ions (**Figure** **4**). Seven metal ions including Ca^2+^, K^+^, Mg^2+^, Mn^2+^, Fe^2+^, Ni^2+^, and Na^+^ with varied concentrations were tested. Both Tb–Man–Geln and Eu–Man–Geln hydrogels showed different degrees of fluorescence quenching upon metal ions (Figure S14 and Figure 15, Supporting Information), among which, Fe^2+^ ion had the maximal fluorescence quenching efficiency. In addition, with the increase of the Fe^2+^ concentration, the fluorescence intensity of Tb–Man–Geln and Eu–Man–Geln hydrogels showed continuous decrease (Figure 4c,d). The fluorochromic response of Tb–Man–Geln and Eu–Man–Geln hydrogels upon Fe^2+^ ion was reversible by adding EDTA based on the chelate effect of EDTA with Fe^2+^, during which process, the coordination between mannose and lanthanide was strengthened, and the “antenna effect” was recovered. The coordination and dissociation of mannose/lanthanide in Ln–Man (Tb–Man) was monitored by FTIR. As shown in Figure S16 in the Supporting Information, the peaks of mannose at 3411 cm^−1^ (ν_‐OH_) shifted to 3446 cm^−1^ upon the addition of Fe^2+^, and shifted back to 3424 cm^−1^ upon the addition of EDTA, indicating the dissociation and recoordination between ‐OH of mannose and lanthanide. The amount of Fe in the Ln–Man–Geln (Tb–Man–Geln) supernatant after adding Fe^2+^ (*m*_1_) and after adding EDTA (*m*_2_) was quantified by spectrum analysis of inductively coupled plasma (ICP). Compared to the initial added amount of Fe^2+^ (m_0_ = 11.2 µg), *m*_1_ decreased to 2.09 µg, indicating the binding replacing of lanthanide by Fe^2+^ and the dissociation of mannose/lanthanide; while *m*_2_ recovered to 5.36 µg, indicating the formation of Fe^2+^–EDTA and the recoordination of mannose/lanthanide. We further optimized the concentration of Fe^2+^ and EDTA to quench and recover the fluorescence of Tb–Man–Geln and Eu–Man–Geln hydrogels (Figure S17, Supporting Information), realizing the reversible fluorochromic “On/Off” switch. As demonstrated in Figure 4b, we displayed the dynamic fluorochromic response by fabricating Tb–Man–Geln and Eu–Man–Geln hydrogels in test tubes, respectively. Upon adding of Fe^2+^ and EDTA, the fluorochromic “On/Off” switch of Tb–Man–Geln and Eu–Man–Geln hydrogels could be clearly observed. We further prepared Tb–Man–Geln hydrogel in a Petri dish, and demonstrated the reversible process by pressing fingerprint with Fe^2+^ ion and erasing the fingerprint with EDTA (Figure S17b, Supporting Information). The quench‐recovery process of fluorescence intensity of Tb–Man–Geln and Eu–Man–Geln hydrogels was monitored via fluorescent spectra, and the efficiency of recovery reached 88.5% and 85.2%, respectively (Figure 4e,f). The fluorescence intensity was not fully recovered due to the dilution by adding aqueous solution. The fluorochromic “On/Off” switch upon Fe^2+^/EDTA was further examined in cycles, and the recovery efficiency maintained 63.2% and 55.7% after three cycles, respectively (Figure 4g,h). Although there was a decrease in the fluorescence intensity, the “On/Off” switchable emission is reversible in response to Fe^2+^.

Encouraged by the above demonstration of Ln–Man‐Galn hydrogels with remarkable fluorochromic responsiveness upon Fe^2+^/EDTA, we for the first time exploited the application of Ln–Man–Geln hydrogels as fluorochromic matrix for 3D cell culture. Since clear fluorescent visualization of the interactions between the cells and hydrogel during 3D cell culture was meaningful,^13^ it is of great significance to develop a system of dynamically tunable fluorochromic hydrogel, realizing the “On/Off” switch to eliminate and recover the fluorescence of matrix during the observation.

**Figure 5:**
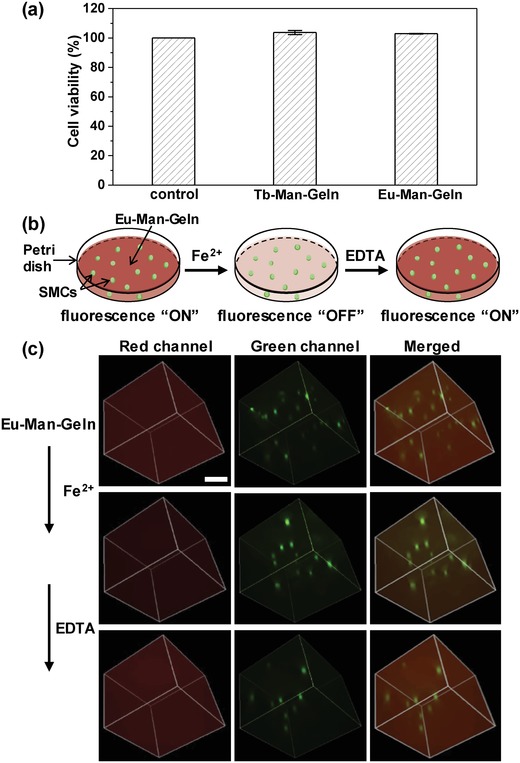
3D cell culture in Ln–Man–Geln hydrogel. a) Cytotoxicity test of Tb–Man–Geln and Eu–Man–Geln hydrogels. b) Illustration of the Eu–Man–Geln hydrogel as a 3D cell culture matrix for reversible fluorochromic “On/Off” switch upon Fe^2+^/EDTA. c) Fluorescence microscope images of Eu–Man–Geln hydrogel as a 3D cell culture matrix for reversible fluorochromic “On/Off” switch upon Fe^2+^ /EDTA observed in red channel, green channel and merged. Scale bar represents 100 µm.

To test the biocompatibility of our hydrogels, we cultured smooth muscle cells (SMCs) with hydrogel extracts and carried out the cytotoxicity test. Both Tb–Man–Geln and Eu–Man–Geln groups showed the similar or even higher cell viability compared with control group, indicating that Tb–Man–Geln and Eu–Man–Geln hydrogels were noncytotoxicity to SMCs (**Figure** **5**a). We then demonstrated the application of 3D cell culture utilizing the Eu–Man–Geln hydrogel, and verified the function of its switchable fluorescence “On/Off” response during the observation of cell culture. The SMCs suspension was added to Eu–Man–Geln hydrogel precursor solution and then resuspended in a Petri dish. Afterward, the cell‐containing Eu–Man–Geln hydrogel was constructed and the SMCs were encapsulated in the hydrogel. The illustration of Eu–Man–Geln hydrogel as a 3D cell culture matrix and its switchable fluorescence “On/Off” response upon Fe^2+^/EDTA during the fluorescence microscopy observation was shown in Figure 5b, and the corresponding images were shown in Figure 5c. The image in red channel exhibited the fluorescence of Eu–Man–Geln hydrogel, and green channel exhibited the SMCs stained with Calcein AM; by merging the two channels, clear visualization of the interactions between the cells and hydrogel was achieved. Furthermore, as a unique system of fluorochromic hydrogel, the fluorescence of Eu–Man–Geln hydrogel could be quenched by adding Fe^2+^ ion and recovered by further adding EDTA. In Figure 5c, the images demonstrated the process of fluorochromic “On/Off” switch of the hydrogel. We expect that this unique fluorochromic “On/Off” system has a potential in the high contrast observation of 3D cell culture with multiple fluorescence labeling: “fluorescence ON” of the hydrogel to verify the position of cells in the matrix; and “fluorescence OFF” of the hydrogel to eliminate the background fluorescence and observe the multistained subcellular structures.

## 3 Conclusion

To summarize, novel lanthanide–mannose complexes were designed and synthesized which showed fluorochromic response to stimuli of Fe^2+^ in solution. Moreover, a gelatin polymeric network was introduced into the lanthanide–mannose complex to fabricate a luminescent hydrogel. The resulting lanthanide hydrogel possessed switchable “On/Off” fluorescence upon Fe^2+^/EDTA, which was realized by the dynamic coordination between lanthanide and mannose controlled by the stimuli of external ions. Furthermore, the fluorochromic hydrogel with good biocompatibility was demonstrated as a 3D cell culture matrix, and realized reversible “On/Off” fluorescence of matrix during the observation of cell culture system. We expect that those stimuli responsive luminescent complexes and hydrogels provide a basic and prospective platform to build functional materials for more applications in biological‐related fields.

## 4 Experimental Section

*Materials*: All commercially reagents and solvents were used without further purification. All of the solvents used were analytical‐reagent grade. Eu_2_O_3_ (99.9%) and Tb_4_O_7_ (99.9%) were purchased from Aladdin. Europium nitrate and terbium nitrate were obtained by dissolving Eu_2_O_3_ and Tb_4_O_7_ in concentrated nitric acid. Glucose, galactose, fructose, sorbose, and mannose were purchased from Solarbio. Gelatin was purchased from Sigma. Iron chloride tetrahydrate (FeCl_2_, >99.7%), magnesium chloride hexahydrate (MgCl_2,_ >98%), manganese chloride (MnCl_2_, >99%), potassium chloride (KCl, >99.5%), sodium chloride (NaCl, >99%), cadmium chloride (CaCl_2_, >74%), and nikel chloride (NiCl_2_, >98.5%) were purchased from Tianjin guangfu science and technology Ltd. NH_4_OH and HCl were purchased from Aladdin.

*Synthesis of the Ln–Man Complexes*: In a typical procedure, a solution of Ln (NO_3_)_3_ (0.1 mol L^−1^) (Ln = Tb, Eu) was added to the solution of ligand (20 wt% mannose) and then by the addition of NH_4_OH until the pH of mixture reached 8.

*Synthesis of the Ln–Man–Geln Hydrogels*: For the fabrication of Ln–Man–Geln hydrogels, Ln–Man complexes were mixed with gelatin solution (18 wt%), and let stood for 10–15 min at room temperature.

*SEM Characterization*: SEM experiments were performed by Hitachi S4800. Samples were put on conducting resin after drying with a freezer dryer, and then the samples were tested after spraying gold.

*Optical Characterization*: Fluorescence emission spectra were recorded by BioTek Synergy/H1 microplate reader at room temperature.

*Rheological Tests*: Rheological tests were carried out on an AR2000 Rheometer (TA Instruments). Strain sweep tests were carried out from 0.1% to 100% at 20 °C and a fixed frequency (1 Hz).

*Stimuli–Response Study*: For Ln–Man complexes, 50 µL metal ion solutions were added into 200 µL Ln–Man complexes, and 50 µL ddH_2_O was added into 200 µL Ln–Man complex as control. For Ln–Man–Geln hydrogels, 200 µL metal ion solutions were added into 800 µL Ln–Man–Geln hydrogels, and 200 µL ddH_2_O was added into 800 µL Ln–Man–Geln hydrogels as control. Each sample had three repetitions.

*Luminescent Lifetime Tests*: The Luminescence decay time of the Ln–Man complexes and Ln–Man–Geln hydrogels was measured on an Edinburgh Instruments FS920P.

*Fluorescence Microscopy Images*: High‐resolution images of Ln–Man–Geln hydrogels and cell culturing were obtained by fluorescence microscopy (Nikon). Samples were placed on Petri dishes.

*Cell Culture*: For the 3D cell culture, 200 µL SMCs solution was added to 1 mL Eu–Man–Geln hydrogel precursor solution and then resuspended in a Petri dish. After several minutes of standing, the cell/hydrogel was constructed and cultured for 24 h at 37 °C in a CO_2_ incubator.

## Conflict of Interest

The authors declare no conflict of interest.

## Supporting information

Supplementary
