# Supplementary material for: A Reversibly Responsive Fluorochromic Hydrogel Based on Lanthanide–Mannose Complex
Source: Adv Sci (Weinh). 2019 Mar 13;6(10):1802112. doi: 10.1002/advs.201802112 (PMC6523369; doi:10.1002/advs.201802112)
Supplement: Supplementary file 1 — Supplementary [file ADVS-6-1802112-s001.pdf]

Copyright WILEY-VCH Verlag GmbH & Co. KGaA, 69469 Weinheim, Germany, 2019.

ADVANCED SCIENCE
Open Access

Supporting Information

for *Adv. Sci.,* DOI: 10.1002/advs.201802112

A Reversibly Responsive Fluorochromic Hydrogel Based on Lanthanide–Mannose Complex

*Ke Meng, Chi Yao, Qianmin Ma, Zhaohui Xue, Yaping Du, Wenguang Liu, and Dayong Yang**

Copyright WILEY-VCH Verlag GmbH & Co. KGaA, 69469 Weinheim, Germany, 2019.

Supporting Information

A Reversibly Responsive Fluorochromic Hydrogel Based on Lanthanide-Mannose Complex

*Ke Meng#, Chi Yao#, Qianmin Ma, Zhaohui Xue, Yaping Du, Wenguang Liu, Dayong Yang**

K. Meng, Dr. C. Yao, Q. Ma, Dr. Z. Xue, Prof. D. Yang

Frontier Science Center for Synthetic Biology and Key Laboratory of Systems Bioengineering (MOE), School of Chemical Engineering and Technology, Tianjin University, Tianjin, 300350, P.R. China.

Prof. Y. Du

School of Materials Science and Engineering &National Institute for Advanced Materials, Center for Rare Earth and Inorganic Functional Materials, Nankai University, Tianjin 300350, P. R. China

Prof. W.G. Liu

School of Materials Science and Engineering, Tianjin Key Laboratory of Composite and Functional Materials, Tianjin University, Tianjin, 300350, P. R. China

# Ke Meng and Chi Yao contributed equally to this work.

* E-mail: dayong.yang@foxmail.com

Experimental Section

Fourier transform infrared spectra (FTIR). FTIR spectra were collected on a Bruker TENSOR27

spectrometer. The dry samples were grinded with spectroscopic grade potassium bromide and then compressed into disks.

Inductively coupled plasma mass spectrometry (ICP). First, 800μL hydrogel was soaked with 200 μL Fe^{2+} solution (10^{-3} M) for 30 min, then the supernatant was sucked out as sample 1; second, the hydrogel was soaked with 200 μL EDTA solution (10^{-4} M) for 30 min, then the supernatant was sucked out as sample 2. The iron content in the supernatant sample 1&2 was quantified by Thermo iCAP 7400 ICP-OES.

Cytotoxicity assays. Smooth muscle cells (SMCs) were seeded into wells of cell culture plate and incubated at 37 °C in CO_2 incubator overnight. First, 10 μL/well leach liquor of Ln-Man-Geln hydrogels was added and was incubated with cells for 24 h. Next, 10 μL 3-(4,5-Dimethylthiazol-2-yl)-2,5-Diphenyltetrazolium Bromide (MTT) solution and 90 μL fresh medium was added to each well, and cultured for another 4 h. Then, the supernatant was removed and 100 μL Formazan solvent was added to each well. After the crystals being dissolved, the absorbance at 490 nm was tested by a BioTek Synergy/H1 microplate reader.

Figures and Captions

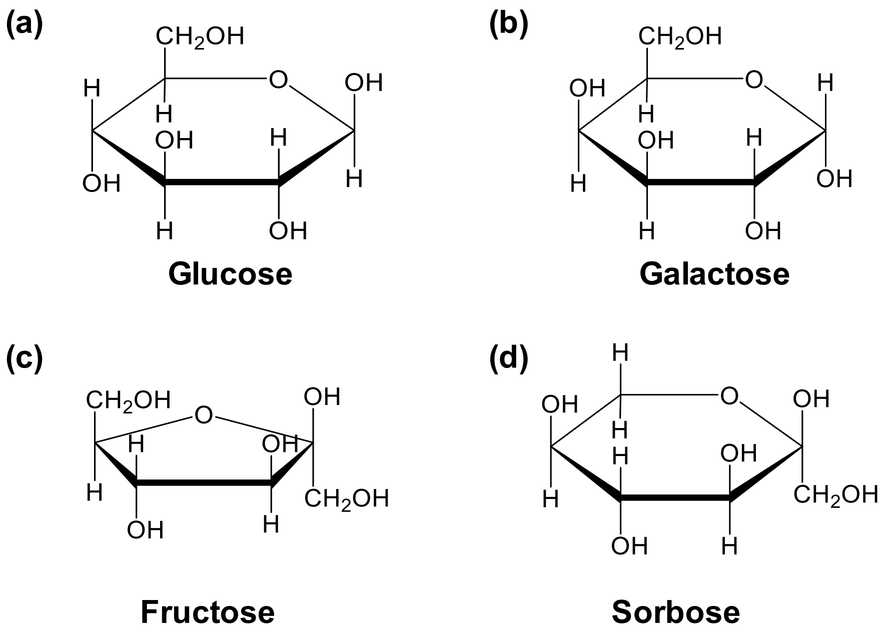

Figure S1. The structure of saccharides: (a) glucose, **(b)** galactose, **(c)** fructose, and **(d)** sorbose.

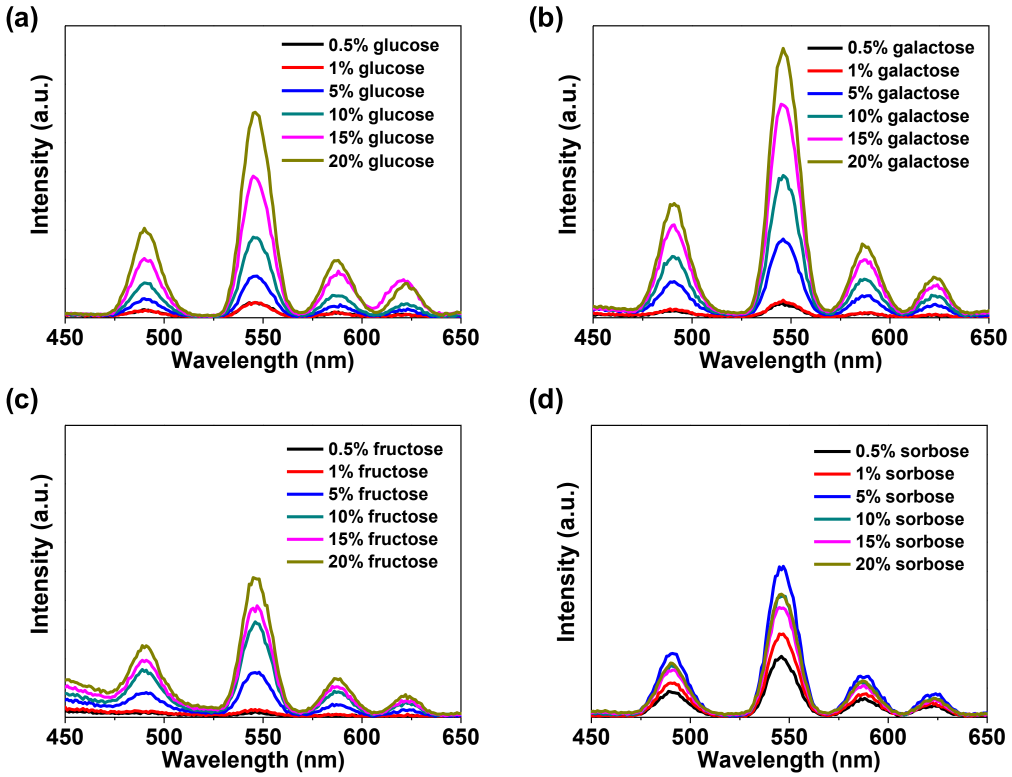

Figure S2. Fluorescent emission spectra of Tb^{3+}-saccharides complexes with different concentration of saccharides upon 312 nm excitation: (a) Tb^{3+}-glucose complex, **(b)** Tb^{3+}-galactose complex, **(c)** Tb^{3+}-fructose complex, and **(d)** Tb^{3+}-sorbose complex.

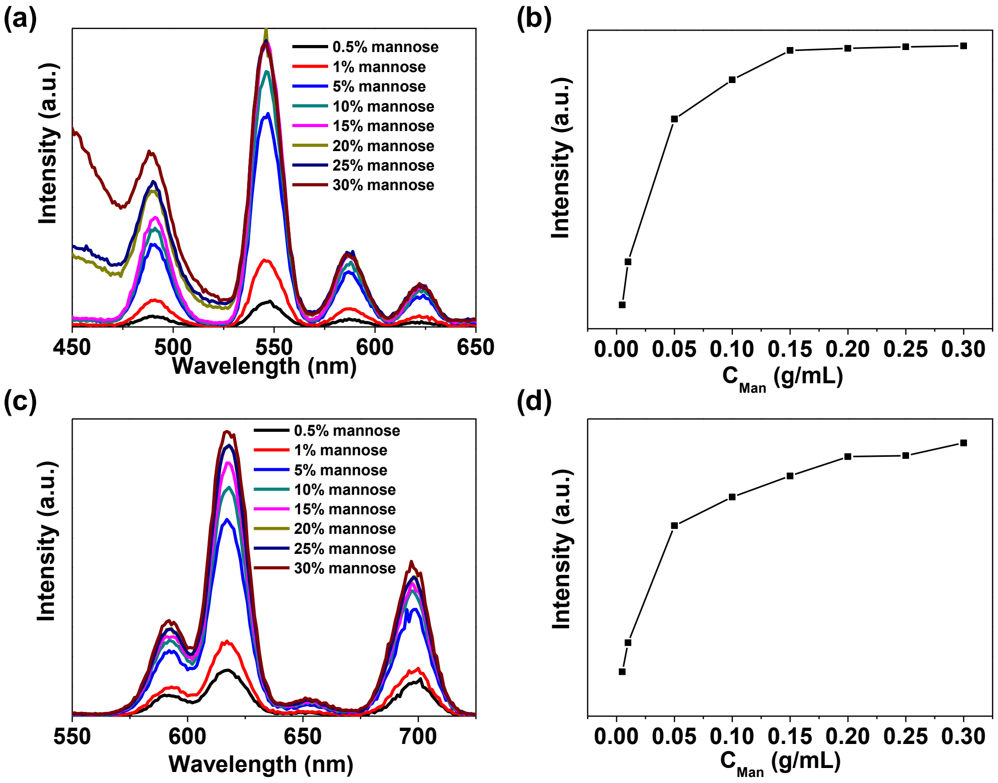

Figure S3. (a) Fluorescent emission spectra of Tb-Man complexes with different concentration of mannose upon 312 nm excitation. **(b)** Fluorescence intensity of Tb-Man complexes at 545 nm. **(c)** Fluorescent emission spectra of Eu-Man complexes with different concentration of mannose upon 312 nm excitation. **(d)** Fluorescence intensity of Eu-Man complexes at 615nm.

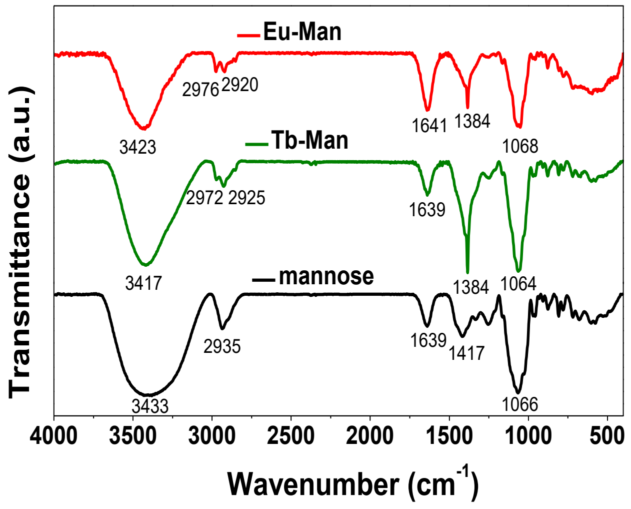

Figure S4. FT-IR spectra of mannose, Tb-Man complex and Eu-Man complex.

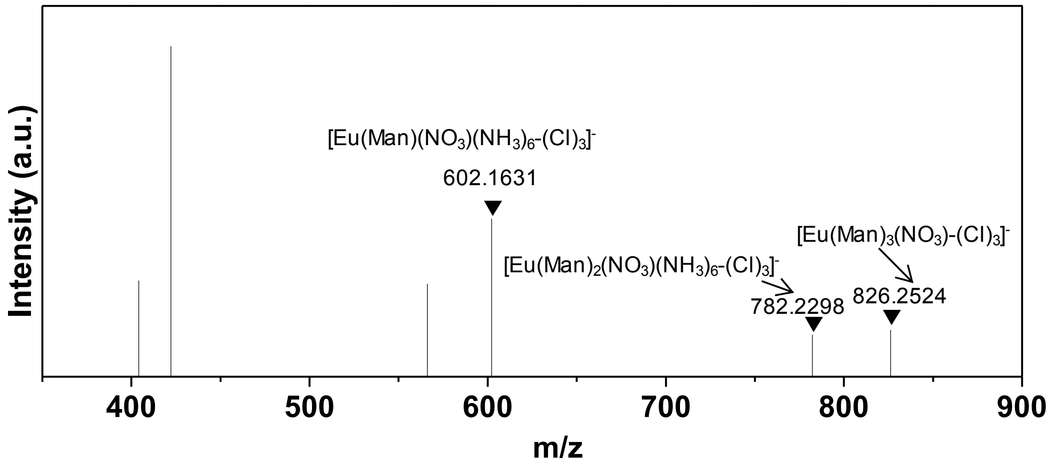

Figure S5. MALDI-MS spectrum of Eu-Man complex.

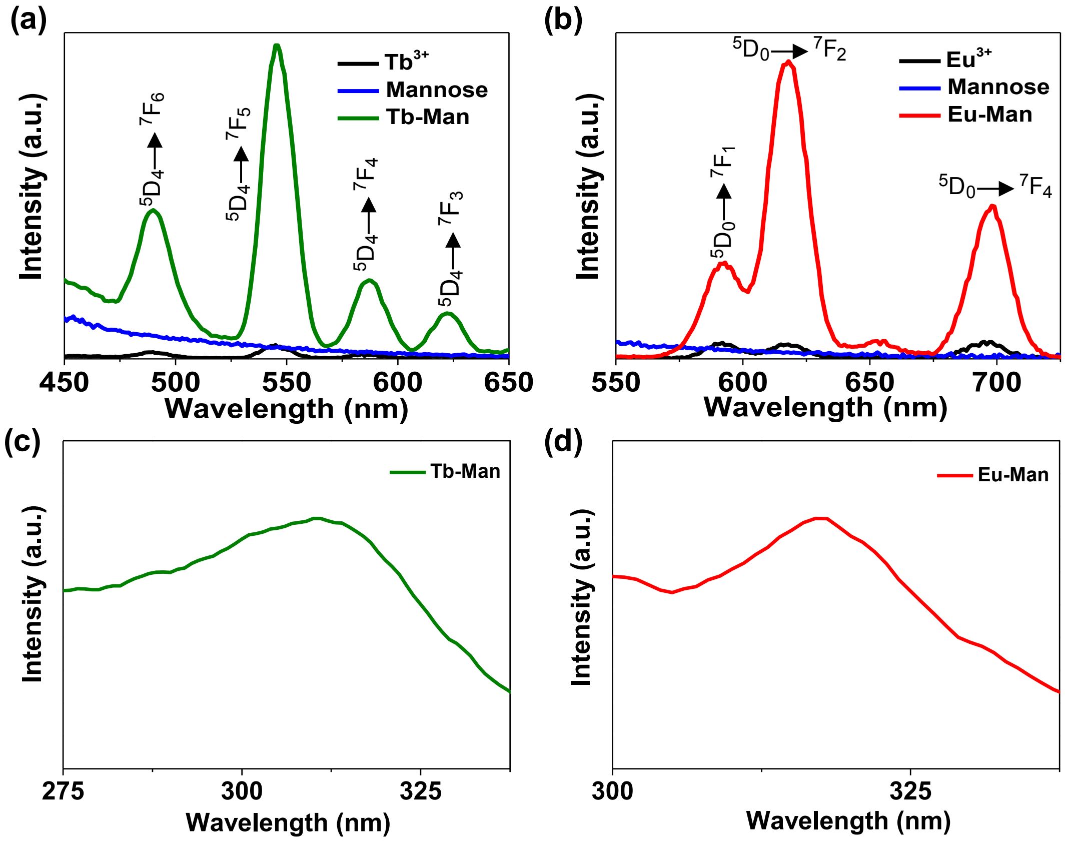

Figure S6. (a&b) Fluorescent emission spectra of Tb^{3+}, mannose and Tb-Man complex (a) and Eu^{3+}, mannose and Eu-Man complex (b). **(c&d)** Fluorescent excitation spectra of Tb-Man complex (c) and Eu-Man complex (d).

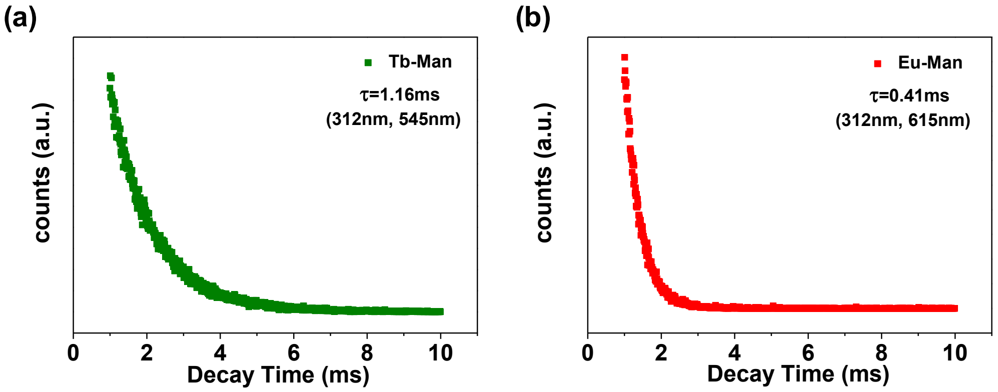

Figure S7. (a&b) The decay curves of Tb-Man complex (a) and Eu-Man complex (b).

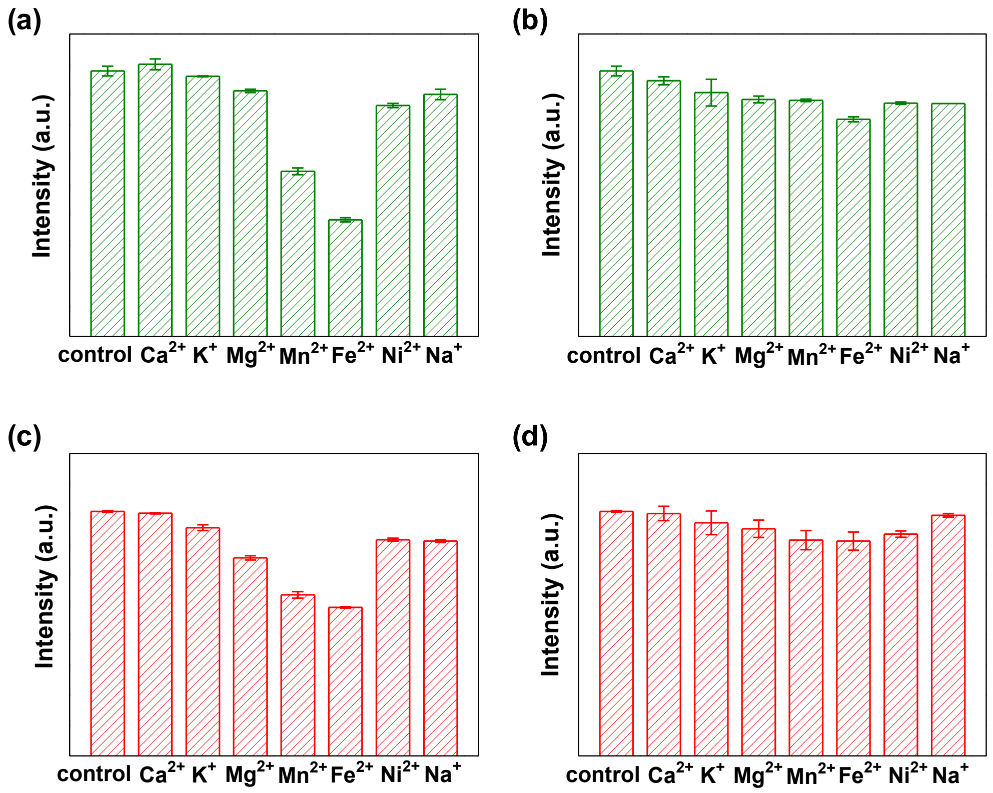

Figure S8. Fluorochromic responses of Tb-Man complex and Eu-Man complex upon metal ions with varied concentration: **(a&c)** 2×10^{-4} M, **(c&d)** 2×10^{-5} M.

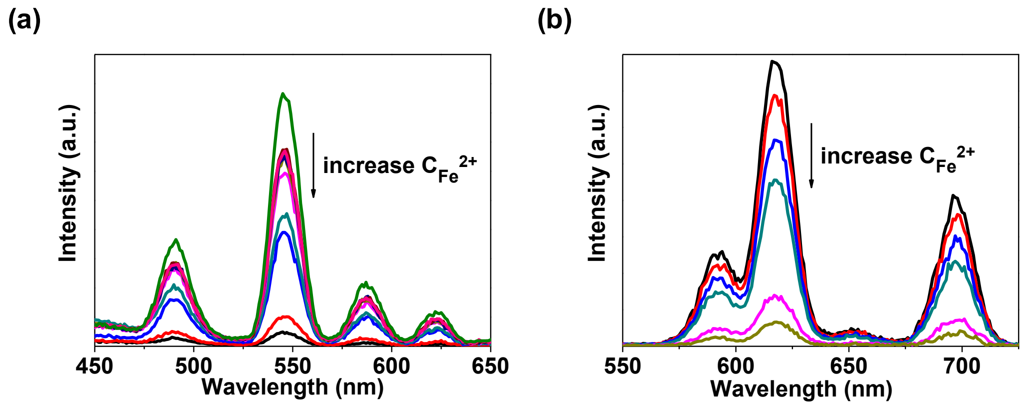

Figure S9. (a&b) Gradual decrease in the fluorescence intensity of Tb-Man complex (a) and Eu-Man complex (b) by increasing the concentration of Fe^{2+} ion.

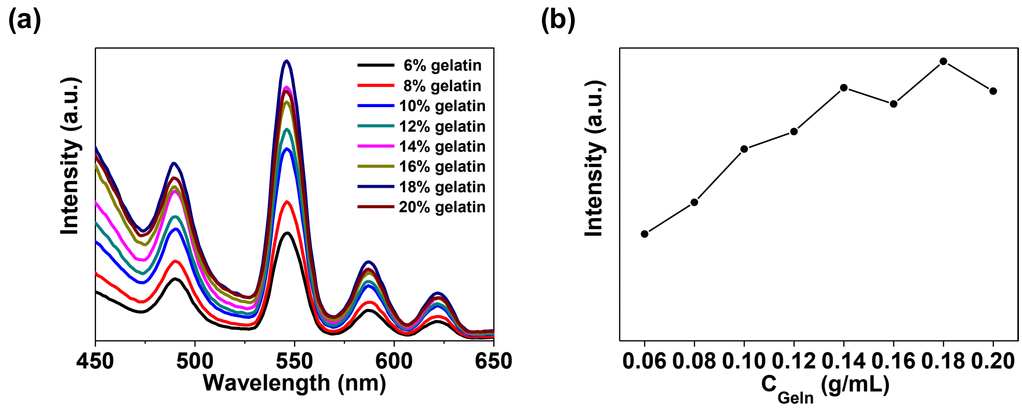

Figure S10. (a) Fluorescent emission spectra of Tb-Man-Geln hydrogel with different concentration of gelatin upon 312 nm excitation. **(b)** Fluorescence intensity of Tb-Man- Geln hydrogel at 545 nm.

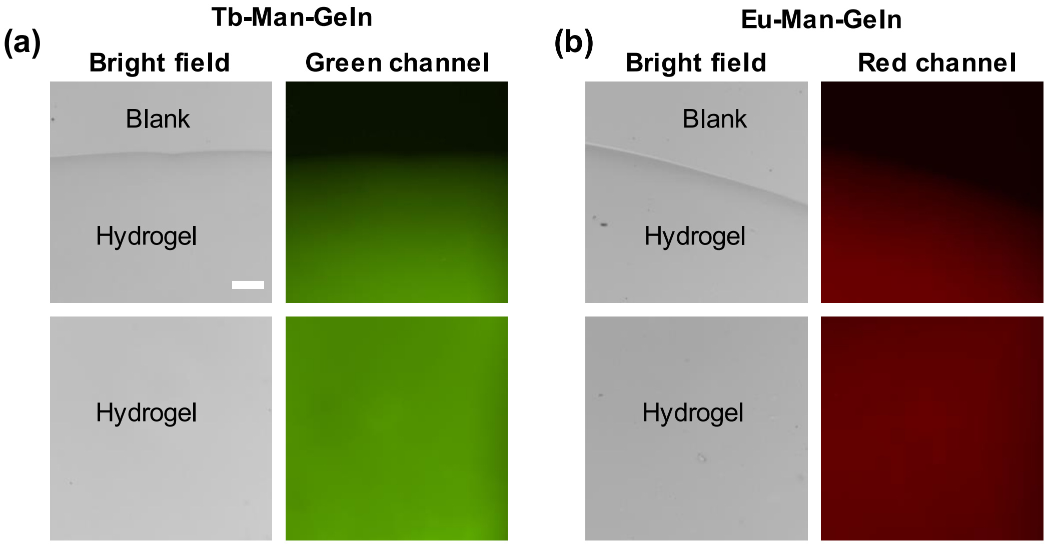

Figure S11. (a&b) Fluorescence microscopy images of Tb-Man-Geln hydrogel (a) and Eu-Man-Geln hydrogel (b). Scale bar represents 60 μm.

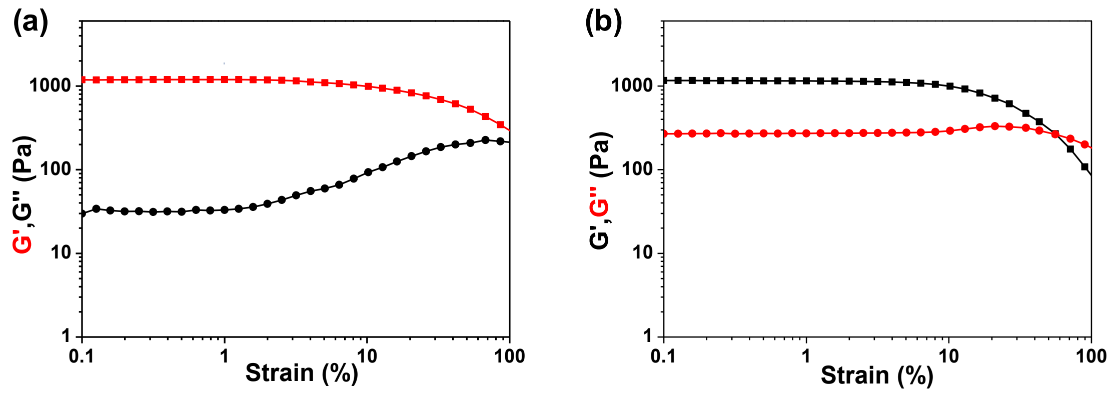

Figure S12. The rheology properties of gelatin (a) and Eu-Man- Geln hydrogel (b).

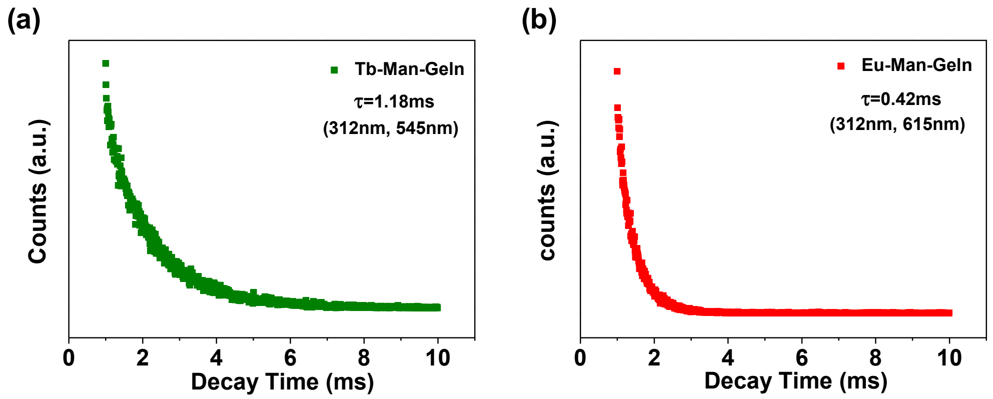

Figure S13. (a&b) The decay curves of Tb-Man-Geln hydrogel (a) and Eu-Man-Geln hydrogel (b).

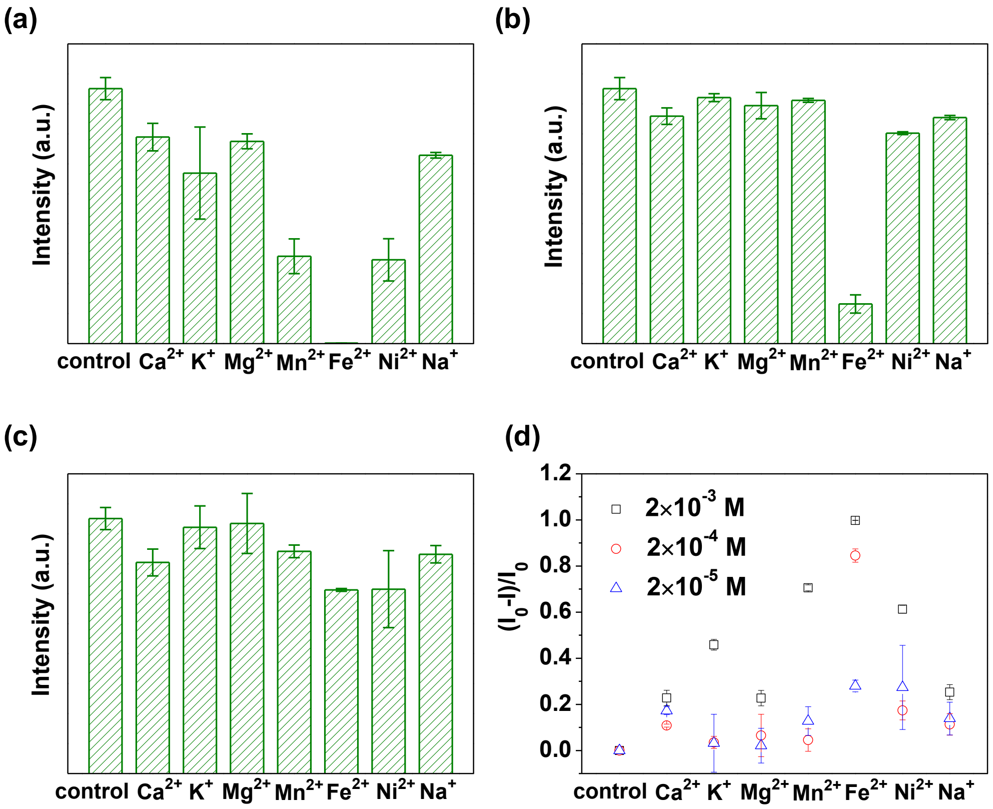

Figure S14. Fluorochromic responses of Tb-Man-Geln hydrogel upon mental ions with varied concentration: **(a)** 2×10^{-3} M, **(b)** 2×10^{-4} M, **(c)** 2×10^{-5} M, **(d)** The quenching efficiency upon mental ions with varied concentration.

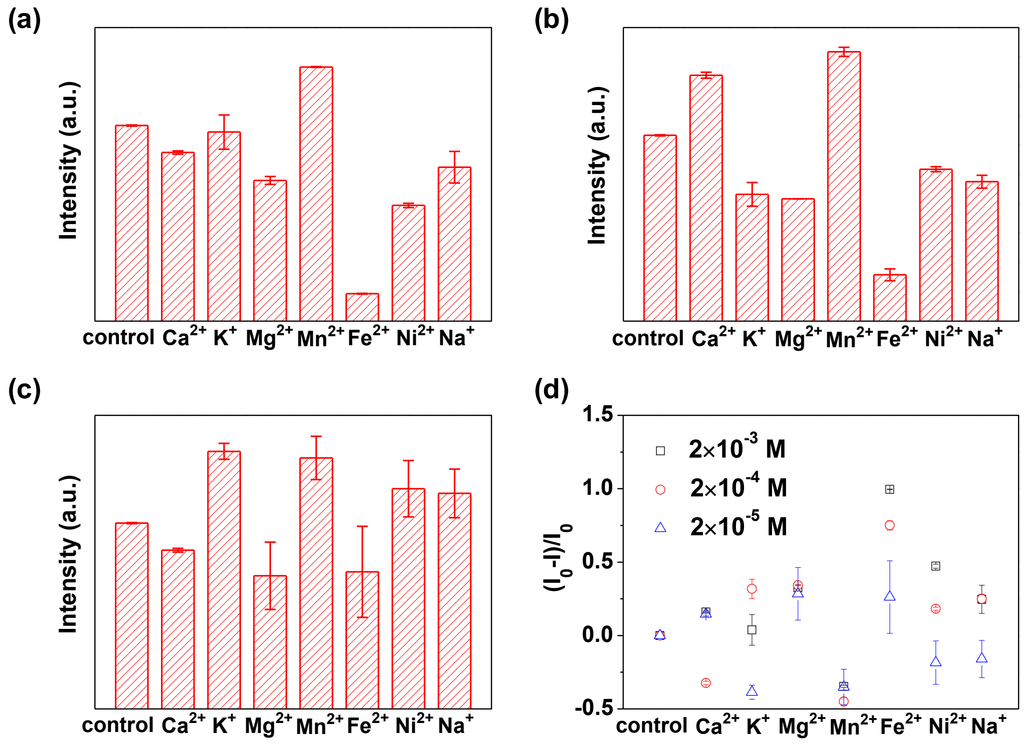

Figure S15. Fluorochromic responses of Eu-Man-Geln hydrogel upon mental ions with varied concentration: **(a)** 2×10^{-3} M, **(b)** 2×10^{-4} M, **(c)** 2×10^{-5} M, **(d)** The quenching efficiency upon mental ions with varied concentration.

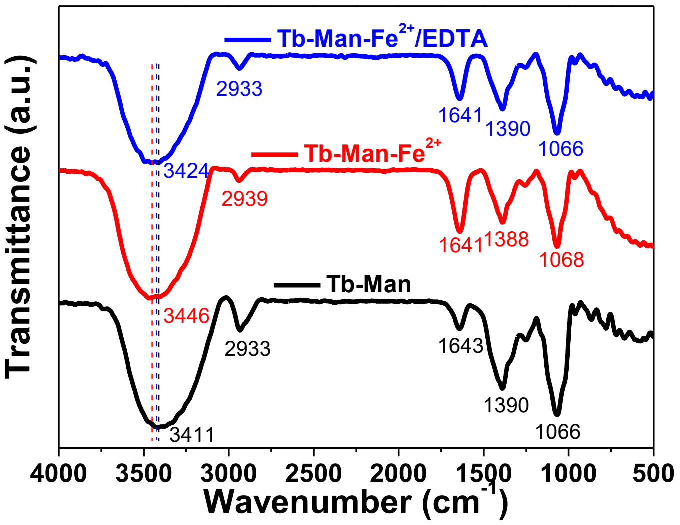

Figure S16. FTIR spectra of Tb-Man complex, upon the addition of Fe^{2+} and upon the addition of EDTA.

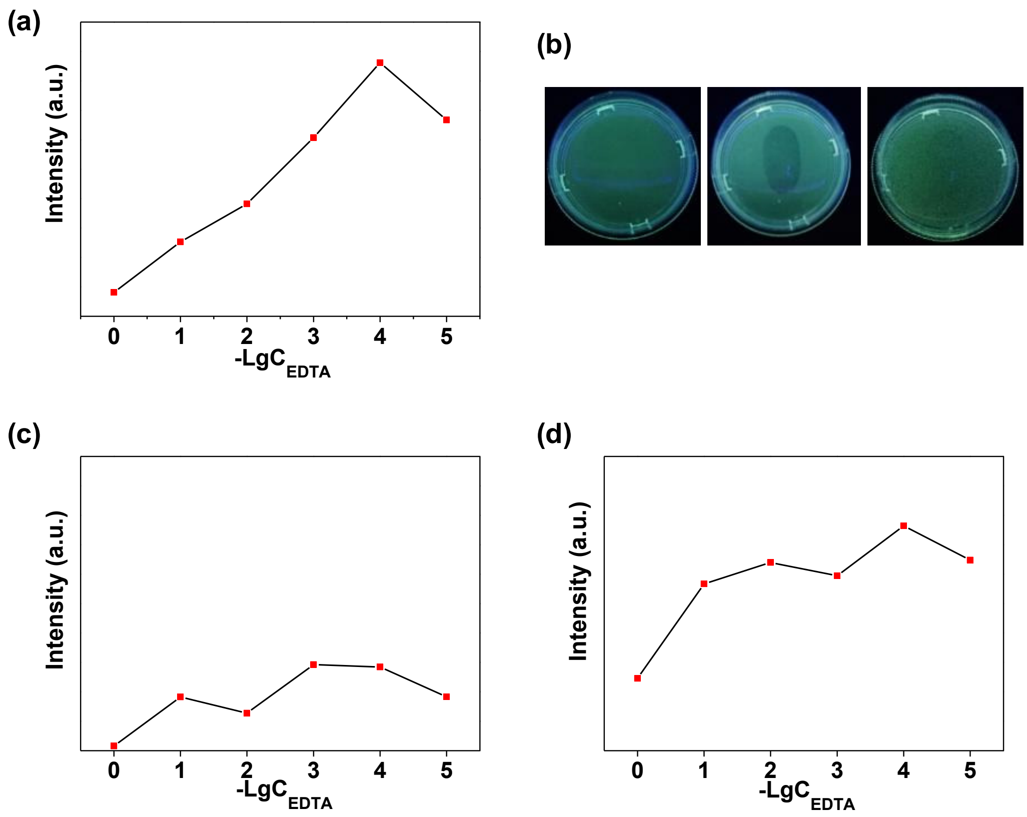

Figure S17. (a) The recovered Tb-Man-Geln hydrogel by adding different concentration of EDTA upon adding Fe^{2+} with final concentration of 2×10^{-4} M. **(b)** The photographs of Tb-Man-Geln hydrogel in a Petri dish under 312 nm UV light: the fluorescence of original hydrogel (left); the fluorescence of hydrogel quenched by pressing fingerprint with Fe^{2+} ion (middle); and the fluorescence of hydrogel recovered by adding EDTA (right). **(c&d)** The recovered Eu-Man-Geln hydrogel by adding varied concentration of EDTA upon adding different final concentration of Fe^{2+}: 2×10^{-3} M (c), 2×10^{-4} M (d).
